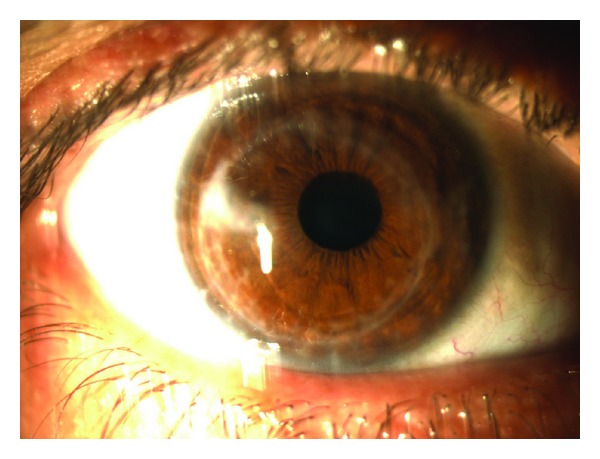# Ipsilateral Rotational Autokeratoplasty for the Management of Traumatic Corneal Scar

**DOI:** 10.1155/2012/853584

**Published:** 2012-10-11

**Authors:** Alime Günes, Tahir Kansu Bozkurt, Cihan Unlu, Betül Ilkay Sezgin Akcay, Hüseyin Bayramlar

**Affiliations:** ^1^Umraniye Training and Research Hospital, Eye Clinic, 34000 Istanbul, Turkey; ^2^Department of Ophthalmology, Medeniyet University Göztepe Training and Research Hospital, 34000 Istanbul, Turkey

## Abstract

A 40-years-old male patient with a corneal scar secondary to perforating eye injury had undergone ipsilateral rotational autokeratoplasty in our clinics. The corneal scar involved the pupillary area. The patient had a preoperative visual acuity of counting fingers. The patient's cornea was trephined with a 0.5 mm temporal decentration. The 8.0 mm autograft was rotated approximately 180° to relocate the scar to the temporal aspect of the cornea. The final position of the corneal scar was temporal of the visual axis and central area was clear. The visual acuity at 1-, 3-, and 6-months followups was better than the first visual acuity in the patient. Ipsilateral rotational autokeratoplasty has many advantages over conventional keratoplasty. There is no risk of immunological rejection of the graft, postoperative corticosteroids are not needed as frequently, and donor cornea is not required. A rotational autograft can be a powerful alternative to conventional keratoplasty for some patients with traumatic corneal scars.

## 1. Introduction

Corneal scars following penetrating corneal injuries cause significant visual reduction. Direct obscuration of rays by the corneal opacity and the irregular corneal astigmatism due to traumatic corneal injury are the reasons for visual impairment [1]. Commonly practiced method of treatment of corneal opacities is penetrating keratoplasty (PK). Traumatic corneal scar appears to be one of the major indications for corneal transplantation in studies from different countries [2–6]. The main problem encountered with this procedure is (early or late) graft rejection, which mandates continued followup of the patients and prompt intervention. In the event of loss of graft clarity due to graft failure, regrafting which brings a higher possibility of graft rejection is required to restore visual function [2].

Autokeratoplasty has been reported as an alternative to PK in selected cases in which the patient has a nonprogressive corneal opacity and a clear cornea in one side [7–12]. Ipsilateral rotational autokeratoplasty (IRA) involves an eccentric trephination of the cornea and “dialing out” the opacity from the visual axis and “dialing in” clear peripheral cornea, thereby creating a clear visual axis. The main advantage of autokeratoplasty is that there is no immunological problem and possibility of graft rejection [10–12]. However, IRA is a treatment choice for very limited number of corneal diseases since many corneal pathologies involve the entire cornea. On the other hand, traumatic corneal scars, which generally originated from an injury with sharp objects or following car or industrial accidents, are generally in a radial fashion and eventually form linear scars. With the given advantages, IRA may be a good alternative to PK in such cases.

In this case report, we describe the surgical procedure and the results of IRA in a patient with a corneal scar secondary to penetrating eye injury. 

## 2. Case Report

A 40-year-old male patient applied to our clinics for low vision in the right eye for one year. He had corneal saturation due to a stone hit into that eye, and the trauma resulted in central corneal scar. The patient had a visual acuity of counting fingers. Slit lamp examination showed a 3 × 2 mm corneal scar involving the pupillary area. The scar extended from nasal quadrant to the center of the cornea. B-mode ultrasound showed no retinal abnormality. His left eye was normal with 10/10 vision. The patient had given a written informed consent, and we had performed IRA. 

## 3. Ipsilateral Rotational Autokeratoplasty

**Surgical Technique**

Under retrobulbar anesthesia, central cornea was marked following routine surgical area cleaning. The recipient cornea involving scar tissue was trephined with a 8 mm punch trephine in a 0.5 mm temporally decentralized fashion. The acquired autograft was rotated about 180 degrees. We tried to clear the pupillary area by placing the scar tissue temporally. The autograft was sutured to recipient cornea with continuous sutures.

Postoperatively, the patient received ofloxacin 0.3%, prednisolone acetate 1%, and preservative-free artificial tears eye drops 4 times a day for one month. Artificial tears and prednisolone acetate 1% eye drops were tapered off over 2 months.

The final position of the corneal scar was temporal of the visual axis, and central area was clear (Figure 1). The spectacle-corrected Snellen visual acuity at postoperative 1 year was 0.6. At the final visit, spherical equivalent was 3.5 diopter (D), and the patient had an astigmatism of 4.5 D. The mean keratometer value was 38.87.

## 4. Discussion

This patient had low vision in the right eye due to a traumatic corneal scar, and there was a significant improvement in the patient's vision that occurred after IRA. The commonly practiced surgical technique for corneal opacities is PK. The main problem is difficulty to acquire donor corneas and the resulting long list of patients waiting for surgery. The other problem encountered with this procedure is graft rejection. Even cornea is an avascular tissue and there is no need for systemic immunosuppression, regrafts are one of the major indications of corneal transplantation [2–6]. Moreover, traumatic scars may be prone to corneal vascularization, and anatomy of the anterior segment following penetrating injuries of cornea (e.g., with iris damage causing peripheral anterior synechia) may lead to graft rejection.

IRA has many advantages over PK. First, autokeratoplasty necessitates no donor cornea and can be performed in places where donor corneas are not readily available [13]. Secondly, there is no risk of immunological rejection. Thus, postoperative corticosteroids are not needed as frequently, and complications related to long-term steroid use such as cataract formation and increased intraocular pressure are significantly decreased when compared to PK.

The main step for success in IRA is the patient selection. Appropriate patient selection depends on prediction of the appearance of graft cornea (and location of the corneal scar) following surgery. In the literature various methods were described to achieve good results after surgery. In summary, these methods are surgical guides, mathematical formulas, and digital image processing [10, 13, 14]. Afshari et al. reported that good results may be achieved in most of the cases by using a 8 mm graft with a 0.5 mm decentralization [13]. We used the same method for our patient. Digital corneal image and computer software were used to plan the graft size and centralization [14]. By adjusting graft size, location, and amount of rotation, centralization of clear cornea at pupillary axis and scar location for best cosmetic result can be planned.

Besides many advantages, IRA has some disadvantages. Generally, IRA may not provide as good visual rehabilitation as PK and may result in higher astigmatism [15]. Potential reasons for the increased astigmatism, if this truly occurs, are the eccentric trephination, disparity of corneal thickness between the peripheral clear cornea and the central scarred cornea into which it is sutured, and the proximity of one edge of the trephination to the corneal pupillary zone [11]. However, there is always a second chance. Autokeratoplasty history is not a drawback for performing PK. 

In conclusion, good results may be achieved with appropriate patient selection and surgical planning in IRA. A rotational autokeratoplasty can be a good alternative to conventional PK for selected patients with traumatic corneal scars.

## Figures and Tables

**Figure 1:**